# Oral Health Values, Oral Hygiene Habits, and Preventive Dental Attendance Among Health-Related and Non-Health-Related University Students: A Cross-Sectional Study

**DOI:** 10.3390/dj14070410

**Published:** 2026-07-06

**Authors:** Klara Dulić, Marija Čandrlić, Ivan Miškulin, Kristina Kralik, Davor Jurlina, Katarina Major Poljak, Ingrid Kovačević, Dora Dragičević Tomičić, Emanuela Ham, Slavko Čandrlić

**Affiliations:** 1 Community Healthcare Center of Osijek-Baranja County, Park Kralja Petra Krešimira IV. 8, 31000 Osijek, Croatia; 2 Faculty of Dental Medicine and Health Osijek, Josip Juraj Strossmayer University of Osijek, Crkvena 21, 31000 Osijek, Croatia; 3 Faculty of Medicine Osijek, Josip Juraj Strossmayer University of Osijek, Josipa Huttlera 4, 31000 Osijek, Croatia

**Keywords:** oral health, oral hygiene, health behavior, students, preventive dentistry, health knowledge, attitudes, practice, surveys and questionnaires

## Abstract

**Background/Objectives:** This study aimed to assess oral health values, oral hygiene habits, preventive dental attendance, and lifestyle-related risk factors among students at the University of Osijek and to compare findings between students enrolled in health-related and non-health-related study programs. **Methods:** A cross-sectional survey was conducted among 331 students (186 health-related and 145 non-health-related). Participants were recruited using a convenience sampling approach. Data were collected using an anonymous questionnaire comprising demographic information, oral health–related behaviors, and the Croatian version of the Oral Health Values Scale (OHVS-CRO). Group differences were analyzed using nonparametric statistical tests. **Results:** Students enrolled in health-related study programs reported significantly more favorable oral hygiene behaviors, including more frequent toothbrushing, greater use of dental floss, interdental brushes, and mouthwash, as well as more regular preventive dental attendance (all *p* < 0.05). They also achieved significantly higher OHVS-CRO scores across all domains and on the total scale (median total score: 42 vs. 40; *p* < 0.001). No significant differences were observed regarding smoking, alcohol consumption, or refined sugar intake. **Conclusions:** Students enrolled in health-related study programs demonstrated higher oral health values and more favorable oral health-related behaviors than students enrolled in non-health-related study programs. These findings suggest an association between educational orientation and oral health values and behaviors and may inform future oral health promotion initiatives targeting university students.

## 1. Introduction

Oral health is an integral component of general health and an important determinant of an individual’s quality of life. Poor oral health can negatively affect nutrition, speech, smile aesthetics, and self-confidence, as well as social relationships and the ability to participate in everyday activities. Despite advances in preventive dentistry, dental caries, periodontal diseases, and other chronic oral conditions remain among the most prevalent diseases worldwide [1]. Education on the importance of oral health and the maintenance of good oral hygiene habits, together with regular use of professional dental services, are key to preventing the onset and progression of oral diseases.

Oral health–related behaviors do not arise solely from the availability of dental services, but also from a set of beliefs, attitudes, and behaviors that individuals attribute to oral health. The concept of oral health values encompasses the perceived importance of maintaining oral health, as well as a willingness to invest time, effort, and financial resources in prevention and treatment [2]. Individuals who place a higher value on oral health are more likely to brush their teeth regularly, use auxiliary oral hygiene aids, maintain healthy dietary habits, and seek dental care in a timely manner [3]. Furthermore, oral health values are closely related to the concept of oral health literacy, which refers to an individual’s ability to obtain, understand, and use oral health information to make appropriate health-related decisions. Higher levels of oral health literacy have been associated with more favorable oral hygiene behaviors, increased use of preventive dental services, and greater engagement in self-care practices [4,5,6].

To better understand and quantify oral health values, standardized scales have been developed. Among them, the Oral Health Values Scale (OHVS) has emerged as a practical and validated instrument for assessing the perceived importance of oral health at the individual level. The OHVS includes several domains, such as professional dental care, aesthetic integrity and tooth preservation, and dental flossing; higher scale scores reflect greater importance attributed to oral health by the respondent [2,3,7]. The scale has been applied in diverse populations and cultural settings and has proven useful in studies examining oral health behaviors and attitudes [8,9,10]. In Croatia, the scale has been adapted and translated for use in clinical and scientific research [11].

From a behavioral perspective, oral health values may represent an important motivational factor linking knowledge and health-related actions, influencing the adoption and maintenance of preventive behaviors throughout adulthood. Hence, the student population is of particular interest for investigating oral health–related values and habits. The period of study is characterized by major life changes, increased independence, and the adoption of long-term health behaviors. Previous research in student populations suggests that students in health-related programs generally demonstrate better oral hygiene habits, greater oral health knowledge, and more frequent use of preventive dental services than students in non-health-related programs [12,13,14]. However, findings are not entirely consistent across different populations and cultural settings. While some studies reported substantial differences in oral health attitudes and behaviors between educational groups, others found only modest differences or identified persistent misconceptions even among health-related students [12,14]. These inconsistencies suggest that educational background alone may not fully explain oral health-related behaviors and that additional determinants, including oral health literacy, personal values, and lifestyle-related factors, should be considered. In addition, most previous studies have focused primarily on oral health knowledge, attitudes, or hygiene practices [9,10]. Oral health values represent a broader construct that reflects the importance individuals attribute to oral health and their willingness to invest effort and resources in its maintenance. Simultaneous assessment of oral health values, oral hygiene habits, utilization of dental services, and lifestyle-related risk factors may provide a more comprehensive understanding of the determinants of oral health behavior among young adults.

Therefore, this study aimed to assess oral health values among students at the University of Osijek using the Croatian version of the Oral Health Values Scale (OHVS-CRO), alongside an evaluation of oral hygiene habits, dental attendance patterns, and lifestyle-related risk factors. Although similar comparisons have been conducted in other countries [14,15], data regarding oral health values among Croatian university students remain scarce, particularly studies utilizing a validated instrument specifically designed to assess oral health values. The present study therefore contributes novel data from a Croatian university population and provides additional evidence regarding the relationship between educational background and oral health-related values and behaviors. We hypothesized that students enrolled in health-related study programs would demonstrate higher oral health values, more favorable oral hygiene habits, and more regular use of preventive dental services compared with students enrolled in non-health-related study programs.

## 2. Materials and Methods

### 2.1. Study Design and Population

The study was designed as a cross-sectional survey and conducted among students at the University of Osijek. It was developed to assess oral health habits and values within a single questionnaire and to examine differences between students enrolled in health-related and non-health-related study programs. The classification of students into health-related and non-health-related study programs was based on the educational orientation of their curricula. Health-related programs include formal education in health sciences, disease prevention, and health promotion, whereas non-health-related programs do not provide comparable health-focused training. The health-related student group included students enrolled in health-related study programs at the Faculty of Dental Medicine and Health (including Dental Medicine, Nursing, Physiotherapy, and related health sciences programs) as well as students from the Faculty of Medicine. The non-health-related group included students from the Faculty of Economics, the Faculty of Electrical Engineering, Computer Science and Information Technology, the Faculty of Agrobiotechnical Sciences, the Faculty of Education, and the Academy of Arts and Culture. Students aged 18 to 30 years were included. Eligibility criteria included:

- Enrollment at the University of Osijek;
- Age between 18 and 30 years;
- Voluntary participation in the study.

The sample was formed using a convenience sampling approach. The questionnaire was distributed through teaching activities and student communication channels. As participation was voluntary and the survey was openly disseminated, the exact number of students who received or viewed the questionnaire could not be determined; therefore, a formal response rate was not calculated. Prior to participation, respondents were provided with an oral or written explanation of the study objectives, the questionnaire content, and the procedures for maintaining data confidentiality. Participation was voluntary and anonymous, and respondents could withdraw at any time without any negative consequences. Only fully completed questionnaires were included in the final analysis. No exclusions were applied based on chronic systemic diseases, previous dental treatment history, or previous dental education, as the aim of the study was to evaluate oral health values and behaviors within a general university student population. In total, 331 respondents were included in the analysis, of whom 186 (56.2%) were students in health-related programs and 145 (43.8%) were students in non-health-related programs. The achieved sample size was considered adequate for the primary comparison between students enrolled in health-related and non-health-related study programs.

### 2.2. Ethical Considerations

The study was approved by the Ethics Committee of the Faculty of Dental Medicine and Health Osijek, J.J. Strossmayer University of Osijek (Class 602-01/25-12/03; No.: 2158/97-97-09-25-09; approved on 13 February 2025). All procedures were conducted in accordance with the ethical standards of the institutional research committee and the principles of the Declaration of Helsinki. Participation was voluntary and anonymous, and all participants were informed about the purpose of the study before completing the questionnaire.

### 2.3. Data Collection Instruments

Data were collected using a standardized questionnaire consisting of three main sections. The first section included demographic and socioeconomic information: age, sex, year and study program, place of residence, number of household members, and estimated monthly household income. The second section assessed oral health–related habits, such as toothbrushing frequency, use of dental floss, interdental brushes, and mouthwash, as well as the frequency of dental visits and reasons for attending. The third section comprised the Oral Health Values Scale (OHVS-CRO). The Croatian version of the OHVS (OHVS-CRO) was previously translated, culturally adapted, and psychometrically validated in a Croatian population, demonstrating satisfactory reliability and validity for use in research settings [11].

The OHVS-CRO is the Croatian version of the original Oral Health Values Scale, developed to measure individuals’ values and attitudes toward different aspects of oral health. The scale consists of 12 items grouped into several domains: professional dental care, aesthetic integrity and tooth preservation, and dental flossing. Respondents indicate their level of agreement with each statement on a Likert scale ranging from “strongly disagree” to “strongly agree.” Some items are negatively worded; therefore, their responses are reverse coded during analysis (the highest response is assigned the lowest number of points and vice versa) so that a higher total score consistently reflects higher oral health values. Total scores range from 12 to 60 points, and separate scores can also be calculated for individual domains. Items marked with an asterisk in the questionnaire were reverse scored prior to analysis. Domain scores were calculated by summing the corresponding item scores within each domain, whereas the total OHVS-CRO score was obtained by summing all 12 item scores. Higher scores indicate greater importance attributed to oral health.

### 2.4. Statistical Analaysis

Statistical data analysis was performed using statistical software MedCalc^®^ Statistical Software v. 23.2.8 (MedCalc Software Ltd., Ostend, Belgium). Only fully completed questionnaires were included in the final analysis; therefore, no imputation procedures for missing data were required. Categorical variables were presented as absolute and relative frequencies, whereas continuous variables were summarized using the median and interquartile range due to deviations from a normal distribution. The Shapiro–Wilk test was used to assess the normality of continuous variables. Differences between two independent groups (health-related and non-health-related students) were analyzed using the Mann–Whitney U test, while comparisons across multiple groups were performed using the Kruskal–Wallis test followed by Conover post hoc comparisons when appropriate. The level of statistical significance was set at *p* < 0.05. Given the exploratory nature of the study, no formal adjustment for multiple comparisons was applied. Internal consistency of the OHVS-CRO scale and its domains was assessed using Cronbach’s alpha coefficient, with values above 0.7 considered acceptable.

## 3. Results

### 3.1. Participant Characteristics

A total of 331 students participated in the study, including 186 (56.2%) students enrolled in health-related study programs and 145 (43.8%) students enrolled in non-health-related study programs. Female participants predominated in both groups (71.0%). No significant differences were observed between groups regarding age, sex distribution, household size, or monthly household income. Significant differences were observed in year of study (*p* < 0.001) and place of residence (*p* = 0.007) between the study groups. Participant characteristics are presented in Table 1.

### 3.2. Oral Hygiene Practices and Use of Preventive Dental Services

Significant differences were observed between students enrolled in health-related and non-health-related study programs regarding oral hygiene practices and utilization of preventive dental services (Table 2). Students from health-related programs reported more favorable oral hygiene behaviors, including more frequent tooth brushing and greater use of mouthwash, dental floss, and interdental brushes. They also attended preventive dental check-ups more regularly, whereas students from non-health-related programs more frequently reported visiting a dentist only when experiencing pain or oral discomfort.

### 3.3. Smoking, Alcohol Consumption, and Refined Sugar Intake

No significant differences were observed between students enrolled in health-related and non-health-related study programs regarding smoking status, alcohol consumption, or refined sugar intake (Table 3). Overall, 27.8% of participants reported being regular smokers, while 33.8% reported occasional alcohol consumption (1–2 times per week). Most participants consumed refined sugars no more than once daily (59.5%), whereas frequent consumption (four or more times daily) was reported by only 6.0% of the study population.

### 3.4. Oral Health Values (OHVS)

The overall OHVS-CRO scale demonstrated acceptable internal consistency. While the Aesthetic Integrity and Tooth Preservation domain showed good reliability, lower Cronbach’s alpha values were observed for the Professional Dental Care and Dental Flossing domains (Table 4).

Item-level analysis of the OHVS-CRO scale demonstrated generally positive attitudes toward oral health among the study participants. The highest levels of agreement were observed for statements related to preserving natural teeth, smile aesthetics, and the importance of oral health for overall health. Greater variability in responses was observed for items related to dental flossing practices and certain aspects of professional dental care. Participants’ responses to individual OHVS-CRO items are presented in Table 5.

Descriptive analysis of OHVS-CRO scores showed that the highest median values were observed in the Aesthetic Integrity and Retention of Teeth domain. The median total OHVS-CRO score was 41 (IQR 38–45) (Table 6).

### 3.5. OHVS Scores According to Participant Characteristics

Students enrolled in health-related programs demonstrated significantly higher scores across all OHVS domains and the overall OHVS score compared with students enrolled in non-health-related programs (all *p* ≤ 0.002). Similarly, female participants achieved significantly higher scores than males in all domains and in the total OHVS score (all *p* < 0.001). Monthly household income was associated only with the Flossing domain, with participants from higher-income households reporting significantly higher scores than those from middle-income households (*p* = 0.007). No significant differences were observed for the remaining OHVS domains (Table 7). The observed differences between groups were consistent across all OHVS domains and ranged from 1 to 3 points, with the largest difference observed for the Flossing domain and the total OHVS score.

### 3.6. OHVS Scores According to Health-Related Behaviors

Smoking status was not significantly associated with any OHVS domain or the overall OHVS score. In contrast, alcohol consumption and refined sugar intake were significantly associated with oral health values (Table 8). Participants who reported no or very infrequent alcohol consumption demonstrated higher scores in the Professional Dental Care and Flossing domains, as well as higher overall OHVS scores, compared with those who consumed alcohol more frequently. Similarly, frequent consumption of refined sugars was associated with lower scores across all OHVS domains and the total OHVS score. Participants who consumed refined sugars four or more times daily consistently reported the lowest OHVS scores across all domains and the total score.

## 4. Discussion

The need for a better understanding of oral health values among young people is linked to efforts to improve preventive programs and increase access to educational content. Analyzing differences between students in health-related and non-health-related study programs can help identify groups that require additional support and education. Overall, the findings support the study hypothesis, as students enrolled in health-related study programs demonstrated higher oral health values, more favorable oral hygiene habits, and more regular use of preventive dental services than students enrolled in non-health-related study programs.

The results of this study showed that students in health-related study programs at the University of Osijek report higher oral health values and more favorable oral hygiene habits compared with students in non-health-related programs. Students in health-related programs more often brush their teeth three or more times per day, use dental floss and other auxiliary oral hygiene aids more regularly, and attend preventive dental check-ups more frequently. These findings suggest that educational exposure may play an important role in shaping oral health values and preventive behaviors. However, the observed differences are unlikely to be explained solely by knowledge acquisition. Students who choose health-related study programs may already possess greater health awareness and stronger preventive orientations before entering university. Therefore, the relationship between educational background and oral health values is likely influenced by both formal education and pre-existing personal attitudes toward health.

The findings are consistent with numerous previous studies showing that students of dental medicine and other health-related programs have better oral hygiene habits and more positive attitudes toward oral health than students in non-health-related study programs [12,13,14,16]. In those studies, health-related students more often reported regular preventive check-ups, more frequent use of additional oral hygiene aids, and a greater willingness to invest in their own oral health. Our results further confirm these trends in the Croatian context, using a standardized instrument to measure oral health values.

The use of the OHVS-CRO scale in this study provided a more detailed insight into the different dimensions of the value that students place on oral health. It was found that students in health-related programs achieved higher scores not only on the total scale but also across all individual domains: professional dental care, aesthetic integrity and tooth preservation, and dental flossing. This indicates that health-related students place greater value on professional dental care, the importance of preserving natural teeth and smile aesthetics, and are more inclined to include dental flossing in their daily routine. These findings are consistent with earlier research emphasizing the importance of education and knowledge in shaping oral health values and behaviors [2,7,9]. The present findings may also be interpreted through the framework of oral health literacy. Individuals with higher levels of oral health literacy are generally better equipped to obtain, understand, and apply oral health information when making health-related decisions. Previous studies have demonstrated associations between oral health literacy, preventive behaviors, use of dental services, and oral health outcomes [4,5,6,12,17]. Therefore, the higher OHVS-CRO scores observed among students enrolled in health-related study programs may partly reflect greater exposure to health-related information and a stronger ability to translate knowledge into preventive oral health practices.

An interesting finding is that no significant differences were observed between the groups in the presence of certain risk factors, such as smoking, alcohol consumption, and the frequency of refined sugar intake. Interestingly, this finding differs from several previous studies that reported healthier lifestyle patterns among health-related students [18,19]. These findings suggest that oral health values and oral hygiene behaviors may not necessarily translate into healthier lifestyle choices in all domains. It implies that complexity of health behavior and supports behavioral models suggesting that knowledge alone is often insufficient to produce sustained lifestyle change. Social influences, environmental factors, and established habits may continue to affect behavior even among individuals with greater health-related knowledge [20].

The overall OHVS-CRO scale demonstrated acceptable internal consistency, and the obtained Cronbach’s alpha values were comparable to those reported in other studies that used the original or adapted versions of the OHVS [8,10,21]. Particularly noteworthy is the high reliability of the Aesthetic Integrity and Tooth Preservation domain, indicating stable measurement in an area that is highly relevant to young populations. Aesthetic dimensions of oral health, such as the appearance of the smile and perceptions of one’s own teeth, often have a strong impact on self-confidence and social interaction; therefore, understanding these values is essential for planning educational content.

The results of this study have several important implications for practice and public health. First, they confirm that students in health-related programs serve as a better example regarding adopting desirable oral health–related habits and values. However, the fact that a substantial proportion of students in non-health-related programs exhibit less favorable habits and lower oral health values highlights the need to expand educational activities beyond health-related curricula. The observed differences suggest that oral health promotion initiatives targeting the wider student population may be warranted. Potential approaches could include educational workshops, elective courses, or awareness campaigns; however, the effectiveness of such interventions should be evaluated in future studies [18,19,22,23].

Second, the use of the OHVS-CRO scale proved useful for identifying areas in which oral health values are less strongly expressed. For example, lower levels of agreement in the domain related to regular dental floss use indicate the need for educational programs to emphasize the importance of interdental hygiene, even among students in health-related programs. Similarly, variability in attitudes toward giving up certain dietary habits to preserve oral health suggests that future interventions should also include a nutrition counseling component [17,22,24].

Third, the findings can serve as a basis for planning longitudinal studies that would track changes in oral health–related values and habits throughout the course of study [17,24]. This could help determine the extent to which educational activities and clinical experience contribute to positive behavioral changes, particularly among students in health-related programs. It would also be useful to extend the research to other universities in Croatia to examine the generalizability of these findings.

This study has several limitations that should be considered when interpreting the findings. First, participants were recruited using a convenience sampling approach, which may have introduced selection bias and limits the generalizability of the results to the broader population of Croatian university students. In addition, the unequal sex distribution between study groups may have influenced some of the observed differences, given the known association between sex and oral health attitudes and behaviors. The cross-sectional design precludes conclusions regarding causality; therefore, although students enrolled in health-related study programs demonstrated higher oral health values and more favorable oral health behaviors, it cannot be determined whether these differences resulted from educational exposure or from pre-existing characteristics that influenced study program selection. Furthermore, the study relied on self-reported data, which may be affected by recall bias and social desirability bias, potentially leading participants to report more favorable behaviors than those practiced in everyday life. Another limitation is the absence of objective clinical oral health assessments, which would have enabled comparison between self-reported behaviors and actual oral health status. However, because oral health behaviors and values were assessed using self-reported measures, the findings reflect participants’ perceptions and reported practices rather than objectively verified clinical oral health status. Finally, the analyses were primarily based on univariate comparisons, and potential confounding factors such as sex, socioeconomic status, and place of residence were not adjusted for in multivariable models. Although the observed median difference in total OHVS-CRO score was modest (3 points), the consistency of differences across all domains suggests a meaningful difference in oral health values between educational groups. Future studies should include larger and more representative samples, objective clinical measures, and multivariable analytical approaches to further clarify the determinants of oral health values and behaviors among university students.

## 5. Conclusions

Students enrolled in health-related study programs at the University of Osijek reported higher oral health values and more favorable oral hygiene behaviors compared with students enrolled in non-health-related study programs. They more frequently reported regular toothbrushing, use of interdental cleaning aids, and preventive dental attendance, and achieved higher scores across all OHVS-CRO domains. These findings suggest an association between educational orientation and oral health–related values and behaviors among university students. They also indicate that oral health promotion initiatives targeting students outside health-related study programs may represent a potentially valuable area for future educational and public health efforts.

## Figures and Tables

**Table 1:** Participant characteristics.

Characteristic	Health-Related Programs (n = 186)	Non-Health-Related Programs (n = 145)	Total (n = 331)	*p*-Value *
**Sex, n (%)**				
Male	46 (24.7)	50 (34.5)	96 (29.0)	0.06
Female	140 (75.3)	95 (65.5)	235 (71.0)	
**Age (years), median (IQR)**	23 (21–24)	23 (21–24)	23 (21–24)	0.56
**Year of study, n (%)**				
1st year	32 (17.2)	30 (20.7)	62 (18.7)	<0.001
2nd year	19 (10.2)	41 (28.3)	60 (18.1)	
3rd year	23 (12.4)	43 (29.7)	66 (19.9)	
4th year †	46 (24.7)	10 (6.9)	56 (16.9)	
5th year †	39 (21.0)	21 (14.5)	60 (17.8)	
6th year	27 (14.5)	0 (0.0)	27 (8.5)	
**Place of residence (population size), n (%)**				
<10,000 inhabitants	89 (47.8)	52 (35.9)	141 (42.6)	0.007
10,000–24,999 inhabitants	27 (14.5)	14 (9.7)	41 (12.4)	
25,000–49,999 inhabitants	13 (7.0)	18 (12.4)	31 (9.4)	
50,000–99,999 inhabitants	35 (18.8)	48 (33.1)	83 (25.1)	
≥100,000 inhabitants	22 (11.8)	13 (9.0)	35 (10.6)	
**Household size, n (%)**				
1 member	5 (2.7)	5 (3.4)	10 (3.0)	0.19
2 members	10 (5.4)	14 (9.7)	24 (7.3)	
3 members	36 (19.4)	39 (26.9)	75 (22.7)	
4 members	77 (41.4)	50 (34.5)	127 (38.4)	
≥5 members	58 (31.2)	37 (25.5)	95 (28.7)	
**Monthly household income, n (%)**				
<€1000	14 (7.5)	11 (7.6)	25 (7.6)	0.13
€1000–€2000	49 (26.3)	53 (36.6)	102 (30.8)	
>€2000	123 (66.1)	81 (55.9)	204 (61.6)	

Abbreviations: IQR, interquartile range; * Chi-square test for categorical variables; Mann–Whitney U test for age. † For non-health-related programs, the 4th and 5th years correspond to the 1st and 2nd years of graduate-level study.

**Table 2:** Oral hygiene practices and utilization of preventive dental services according to study field.

Characteristic	Health-Related Programs (n = 186)	Non-Health-Related Programs (n = 145)	Total (n = 331)	*p*-Value *
**Tooth brushing frequency, n (%)**				<0.001
0–1 time/day	8 (4.3)	22 (15.2)	30 (9.1)	
2 times/day	122 (65.6)	100 (69.0)	222 (67.1)	
≥3 times/day	56 (30.1)	23 (15.9)	79 (23.9)	
**Use of additional oral hygiene aids, n (%)**				
Mouthwash	113 (60.8)	61 (42.1)	174 (52.6)	0.001
Dental floss	122 (65.6)	66 (45.5)	188 (56.8)	<0.001
Interdental brushes	84 (45.2)	26 (17.9)	110 (33.2)	<0.001
**Frequency of preventive check-ups, n (%)**				<0.001
Every 6 months	79 (42.5)	34 (23.4)	113 (34.1)	
Once per year	76 (40.9)	58 (40.0)	134 (40.5)	
Only when experiencing pain or discomfort	31 (16.7)	53 (36.6)	84 (25.4)	

* Chi-square test.

**Table 3:** Smoking Habits, Alcohol Consumption, and Refined Sugar Intake According to Study Field.

Characteristic	Health-Related Programs (n = 186)	Non-Health-Related Programs (n = 145)	Total (n = 331)	*p*-Value *
**Smoking status, n (%)**				
Non-smoker	112 (60.2)	81 (55.9)	193 (58.3)	0.71
Occasional smoker (<10 cigarettes/week)	24 (12.9)	22 (15.2)	46 (13.9)	
Regular smoker	50 (26.9)	42 (29.0)	92 (27.8)	
**Alcohol consumption, n (%)**				
Do not drink or drink very rarely	119 (64.0)	92 (63.4)	211 (63.7)	0.19
Occasional consumption (1–2 times/week)	65 (34.9)	47 (32.4)	112 (33.8)	
Frequent consumption (≥3 times/week)	2 (1.1)	6 (4.1)	8 (2.4)	
**Refined sugar consumption, n (%)**				
0–1 time/day	121 (65.1)	76 (52.4)	197 (59.5)	0.06
2–3 times/day	56 (30.1)	58 (40.0)	114 (34.4)	
≥4 times/day	9 (4.8)	11 (7.6)	20 (6.0)	

* Chi-square test.

**Table 4:** Internal consistency of the OHVS domains and total scale.

OHVS Domain	Health-Related Programs	Non-Health-Related Programs	Total Sample
Professional Dental Care	0.588	0.620	0.617
Aesthetic Integrity and Retention of Teeth	0.933	0.917	0.926
Flossing	0.662	0.642	0.688
**OHVS total score**	**0.762**	**0.774**	**0.782**

Abbreviation: OHVS, Oral Health Values Scale.

**Table 5:** Participants’ Responses to Individual OHVS-CRO Items.

OHVS-CRO Item	Strongly Disagree n (%)	Disagree n (%)	Neither Agree nor Disagree n (%)	Agree n (%)	Strongly Agree n (%)
It is important to me to keep my natural teeth.	26 (7.9)	2 (0.6)	33 (10.0)	34 (10.3)	236 (71.3)
* When I do not have time, it is acceptable to skip flossing for a day or two.	60 (18.1)	51 (15.4)	97 (29.3)	67 (20.2)	56 (16.9)
My smile is an important part of my appearance.	23 (6.9)	4 (1.2)	24 (7.3)	62 (18.7)	218 (65.9)
* Going to the dentist is not worth the financial cost to me.	161 (48.6)	93 (28.1)	38 (11.5)	15 (4.5)	24 (7.3)
Flossing my teeth every day is of great importance to me.	51 (15.4)	42 (12.7)	110 (33.2)	72 (21.8)	56 (16.9)
* I would rather wear dentures than spend money on treating dental caries or gum disease.	239 (72.2)	48 (14.5)	24 (7.3)	6 (1.8)	14 (4.2)
It is important to me to be proud of my teeth and gums.	25 (7.6)	8 (2.4)	33 (10.0)	57 (17.2)	208 (62.8)
* If I have a toothache, I would rather wait to see whether it resolves on its own before visiting a dentist.	89 (26.9)	72 (21.8)	70 (21.1)	61 (18.4)	39 (11.8)
* It would not bother me if I had to have an artificial tooth or dentures.	108 (32.6)	84 (25.4)	79 (23.9)	35 (10.6)	25 (7.6)
I make sure that I always have dental floss available when I need it.	107 (32.3)	83 (25.1)	85 (25.7)	29 (8.8)	27 (8.2)
* It is important to me to visit a dentist only when I have problems with my teeth or gums.	84 (25.4)	65 (19.6)	74 (22.4)	56 (16.9)	52 (15.7)
The condition of my teeth and gums is an important part of my overall health.	20 (6.0)	9 (2.7)	33 (10.0)	55 (16.6)	214 (64.7)

* Reverse-scored items. OHVS-CRO = Croatian version of the Oral Health Values Scale.

**Table 6:** Descriptive statistics of OHVS domains and total score.

OHVS Domain	Possible Range	Median (IQR)	Observed Range
Professional Dental Care	5–25	19 (16–21)	5–25
Aesthetic Integrity and Retention of Teeth	4–20	19 (16–20)	4–20
Flossing	3–15	8 (6–10)	3–15
**OHVS total score**	**12–60**	**41 (38–45)**	**23–53**

Abbreviations: OHVS, Oral Health Values Scale; IQR, interquartile range.

**Table 7:** OHVS Scores According to Participant Characteristics.

Characteristic	Professional Dental Care Median (IQR)	Aesthetic Integrity and Retention of Teeth Median (IQR)	Flossing Median (IQR)	OHVS Total Score Median (IQR)
**Study field**				
Health-related programs	20 (17–22)	20 (18–20)	9 (7–11)	42 (39–46)
Non-health-related programs	18 (15–21)	18 (15–20)	7 (5–9)	40 (34–43)
*p*-value *	0.001	0.002	<0.001	<0.001
**Sex**				
Male	17 (15–20)	17 (14–20)	7 (5–9)	39 (33–43)
Female	20 (17–22)	20 (18–20)	9 (7–11)	42 (39–46)
*p*-value *	<0.001	<0.001	<0.001	<0.001
**Monthly household income**				
≤€1000	18 (16–20)	19 (16–20)	8 (7–10)	39 (37–44)
€1001–2000	19 (16–21)	19 (16–20)	8 (6–9)	41 (38–43)
>€2000	19 (17–22)	19 (16–20)	9 (7–11)	42 (38–46)
*p*-value †	0.19	0.96	0.007 ‡	0.06

Abbreviations: OHVS, Oral Health Values Scale; IQR, interquartile range. * Mann–Whitney U test. † Kruskal–Wallis test. ‡ Post hoc analysis (Conover test) showed a significant difference between participants with monthly household incomes of €1001–2000 and >€2000.

**Table 8:** OHVS Scores According to Health-Related Behaviors.

Characteristic	Professional Dental Care Median (IQR)	Aesthetic Integrity and Retention of Teeth Median (IQR)	Flossing Median (IQR)	OHVS Total Score Median (IQR)
**Smoking status**				
Non-smoker (n = 193)	19 (17–21)	19 (17–20)	9 (6–10)	42 (39–45)
Occasional smoker (n = 46)	19 (16–22)	19 (14–20)	8 (6–11)	41 (37–46)
Regular smoker (n = 92)	19 (16–22)	18 (14–20)	8 (7–10)	40 (35–45)
*p*-value *	0.76	0.06	0.99	0.09
**Alcohol consumption**				
No or very rare consumption (n = 211)	20 (17–22)	19 (17–20)	9 (7–11)	42 (39–45)
Occasional consumption (1–2 times/week) (n = 112)	18 (15–21)	19 (15–20)	8 (6–10)	40 (35–45)
Frequent consumption (>3 times/week) (n = 8)	17 (15–22)	16 (12–20)	7 (5–7)	35 (31–41)
*p*-value *	0.04 †	0.06	0.04 ‡	0.005 §
**Refined sugar consumption**				
0–1 time/day (n = 197)	20 (17–21)	20 (17–20)	9 (7–11)	42 (39–45)
2–3 times/day (n = 114)	18 (16–22)	19 (16–20)	8 (6–10)	41 (37–45)
≥4 times/day (n = 20)	17 (15–19)	16 (14–19)	6 (3–9)	35 (32–42)
*p*-value *	0.02	0.007	0.002	0.002

* Kruskal–Wallis test. † Post hoc analysis showed a significant difference between participants reporting no or very rare alcohol consumption and those reporting occasional alcohol consumption. ‡ Post hoc analysis showed a significant difference between participants reporting no or very rare alcohol consumption and those reporting frequent alcohol consumption. § Post hoc analysis showed significant differences between participants reporting no or very rare alcohol consumption and both alcohol-consuming groups. Post hoc analysis showed significant differences between participants consuming refined sugars ≥4 times/day and both lower-frequency consumption groups.

## Data Availability

The data that support the findings of this study are available from the corresponding author upon reasonable request.
